# Urinary Biomarkers KIM-1 and NGAL for Detection of Chronic Kidney Disease of Uncertain Etiology (CKDu) among Agricultural Communities in Sri Lanka

**DOI:** 10.1371/journal.pntd.0004979

**Published:** 2016-09-19

**Authors:** Pallagae Mangala C. S. De Silva, Khaja Shameem Mohammed Abdul, Eakanayake M. D. V. Eakanayake, Sudheera Sammanthi Jayasinghe, Channa Jayasumana, Hewa Bandulage Asanthi, Hettiarachigae S. D. Perera, Gamage G. Tushara Chaminda, Ediriweera P. S. Chandana, Sisira H. Siribaddana

**Affiliations:** 1 Department of Zoology, Faculty of Science, University of Ruhuna, Matara, Sri Lanka; 2 Department of Pharmacology, Faculty of Medicine, University of Ruhuna, Galle, Sri Lanka; 3 Department of Pharmacology, Faculty of Medicine and Allied Sciences, Rajarata University, Saliyapura, Sri Lanka; 4 Department of Limnology, Faculty of Fisheries & Marine Sciences and Technology, University of Ruhuna, Matara, Sri Lanka; 5 Department of Civil Engineering, Faculty of Engineering, University of Ruhuna, Hapugala, Sri Lanka; 6 Department of Medicine, Faculty of Medicine and Allied Sciences, Rajarata University, Saliyapura, Sri Lanka; University of California San Diego School of Medicine, UNITED STATES

## Abstract

Chronic Kidney Disease of uncertain etiology (CKDu) is an emerging epidemic among farming communities in rural Sri Lanka. Victims do not exhibit common causative factors, however, histopathological studies revealed that CKDu is a tubulointerstitial disease. Urine albumin or albumin-creatinine ratio is still being used as a traditional diagnostic tool to identify CKDu, but accuracy and prevalence data generated are questionable. Urinary biomarkers have been used in similar nephropathy and are widely recognised for their sensitivity, specificity and accuracy in determining CKDu and early renal injury. However, these biomarkers have never been used in diagnosing CKDu in Sri Lanka. Male farmers (n = 1734) were recruited from 4 regions in Sri Lanka i.e. Matara and Nuwara Eliya (farming locations with no CKDu prevalence) and two CKDu emerging locations from Hambantota District in Southern Sri Lanka; Angunakolapelessa (EL1) and Bandagiriya (EL2). Albuminuria (ACR ≥ 30mg/g); serum creatinine based estimation of glomerular filtration rate (eGFR); creatinine normalized urinary kidney injury molecule (KIM-1) and neutrophil gelatinase-associated lipocalin (NGAL) were measured. Fourteen new CKDu cases (18%) from EL1 and nine CKDu cases (9%) from EL2 were recognized for the first time from EL1, EL2 locations, which were previously considered as non-endemic of the disease and associated with persistent albuminuria (ACR ≥ 30mg/g Cr). No CKDu cases were identified in non-endemic study locations in Matara (CM) and Nuwara Eliya (CN). Analysis of urinary biomarkers showed urinary KIM-1 and NGAL were significantly higher in new CKDu cases in EL1 and EL2. However, we also reported significantly higher KIM-1 and NGAL in apparently healthy farmers in EL 1 and EL 2 with comparison to both control groups. These observations may indicate possible early renal damage in absence of persistent albuminuria and potential capabilities of urinary KIM-1 and NGAL in early detection of renal injury among farming communities in Southern Sri Lanka.

## Author Summary

Chronic Kidney Disease (CKD) is a challenging global health issue around the world. Impairment of kidney function with time is eminent, but indications of CKD may not be seen until considerable damage to kidney functions. Two main causes of CKD are diabetes and high blood pressure. However recently new form of CKD has been reported among agricultural works in the tropics other than known factors. The causes for this mysterious CKD are unknown and termed as Chronic Kidney Disease of uncertain etiology (CKDu). Clinical diagnosis depends on urinary markers and conventional creatinine based markers may underestimate the prevalence of the disease. Therefore, development of new sensitive markers for the early detection may certainly improve the treatment and patient management around the world.

## Introduction

Chronic Kidney Disease of unknown etiology (CKDu) is an endemic disease among dry zone farming communities in Sri Lanka. First cases were reported in early 1990s in North Central Province (NCP) predominantly among male farmers [1]. It has reached epidemic proportions with ever increasing numbers of patients and deaths, thus becoming a new and emerging health issue that would eventually inflict adverse consequences on food security, merely for the fact that affected populations constitute the major rice farming communities in Sri Lanka. It is a global epidemic as similar types of kidney diseases are reported from Andhra Pradesh in India [2] and in Central America including Nicaragua [3], El Salvador [4] and Costa Rica [5].

Hypertension, diabetes, glomerulonephritis and other traditional causes are not associated with CKDu. However, multiple causes have been suggested such as chronic low dose exposure to multiple heavy metals and agrochemicals [6, 7], heat stress and recurrent dehydration [8–11], heat driven pathophysiologic mechanisms [12], nephrotoxic drugs [13], hyperuricemia and hyperuricosuria [14–17], leptospirosis [18, 19] and genetic susceptibility [20, 21]. Based on clinical and pathological studies, CKDu cases in Sri Lanka show glomerular and tubulointerstitial injury in kidneys [1, 22, 23] and similar glomerular and tubulointerstitial injury have also been reported in Mesoamerican nephropathy [24, 25].

A recent study by the World Health Organization (WHO) has used albumin creatinine ratio (ACR) as the diagnostic criteria for CKDu in Sri Lanka [26]. However, ACR ≥ 30 mg/g Cr may not detect early renal injury [27–29] hence may underestimate disease prevalence. Several novel biomarkers such as human neutrophil gelatinase-associated lipocalin (NGAL), kidney injury molecule 1 (KIM-1), N-acetyl beta glucosaminidase (NAG), interleukin 18 (IL-18), insulin like growth factor-binding protein 7 (IGFBP7) and tissue inhibitor of metalloproteinases-2 (TIMP-2) are being used to diagnose acute kidney injury [29] and their use in chronic kidney disease was evident in Mesoamerican nephropathy [30, 31]. However, tubular markers such as KIM -1 and NGAL have not been used for CKDu diagnosis in Sri Lanka.

KIM-1 also known as T-cell immunoglobulin and mucin-containing molecule is a type 1 trans-membrane protein with molecular weight approximately 100 kDa. Proximal tubular cells in kidneys are the main source of KIM-1 in the urine and it is up-regulated during acute kidney injury [29]. NGAL, also known as siderocalin, lipocalin-2 (lnc2) or lipocalin 24p3 is a 22–25 kDa glycoprotein that belongs to superfamily “lipocalin” [32]. Leucocytes, loop of Henle and collecting ducts are some of the major sources of NGAL in the body [29]. NGAL is released from lysosomes, brush-border and cytoplasm of proximal tubular epithelial cells during chronic kidney disease [29]. Urinary KIM-1 and NGAL are good predictors of renal injury prior to detectable changes in eGFR [33–35]. Moreover, urinary KIM-1 and NGAL are potential biomarkers in predicting chronic kidney disease due to tubulointerstitial damage [36].

The first objective of this study was to determine the prevalence of CKDu using case definition by Jayathilaka et al, [26] mainly focussing on albuminuria (ACR ≥ 30mg/g) and eGFR in disease emerging locations in Hambantota district (EL1 & EL2) and non-endemic areas Matara and Nuwara Eliya (CM & CN) in Sri Lanka. The second objective was to determine the levels of tubular markers KIM-1 and NGAL in the same study populations to assess potential early renal injury among CKDu subjects and healthy farmers from the selected locations.

## Materials and Methods

**Fig 1:**
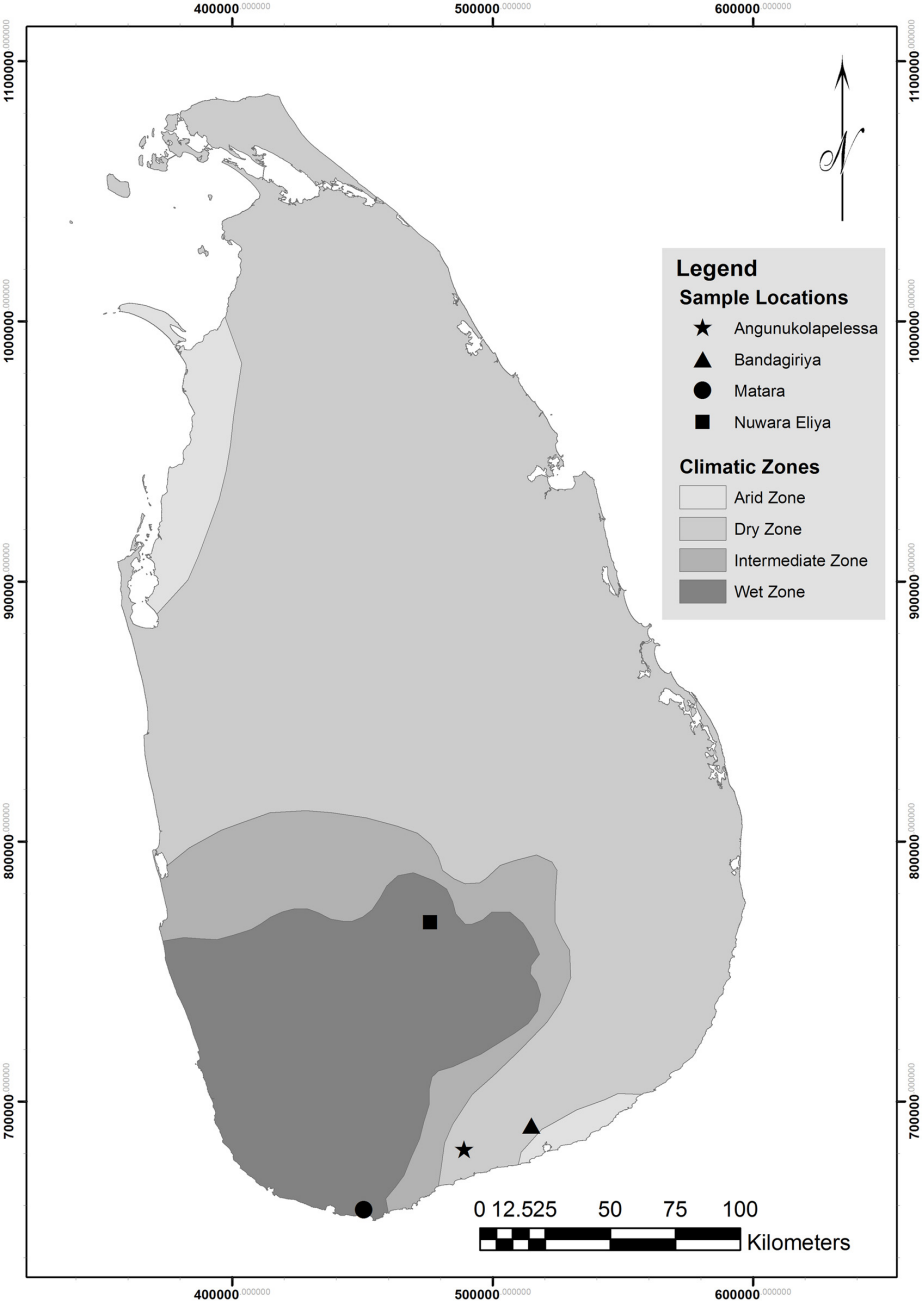
Study locations of the current study (Agunukolapalassa; EL1, Badagiriya; EL2, Matara; CM) in Southern Province and Nuwara Eliya (CN) in Central Province represented with different climatic zones in Sri Lanka.

A cross-sectional study was conducted at three locations namely Angunakolapelessa (EL1), Bandagiriya (EL 2) and Matara (CM) in the Southern Province and one location, Nuwara Eliya (CN) in the Central Province, Sri Lanka (Fig 1). EL1 and EL2 in Hambantota district situated in the dry zone of Sri Lanka (annual mean temperature more than 27.2°C) with similar farming practices to CKDu endemic areas in NCP. New patients with CKDu are recently reported from EL1 and EL2 farming locations. We also recruited male farmers from non-endemic locations in the wet zone, Matara (CM, annual mean temperature more than 27°C) and Nuwara Eliya (CN, annual mean temperature less than 15°C).

**Fig 2:**
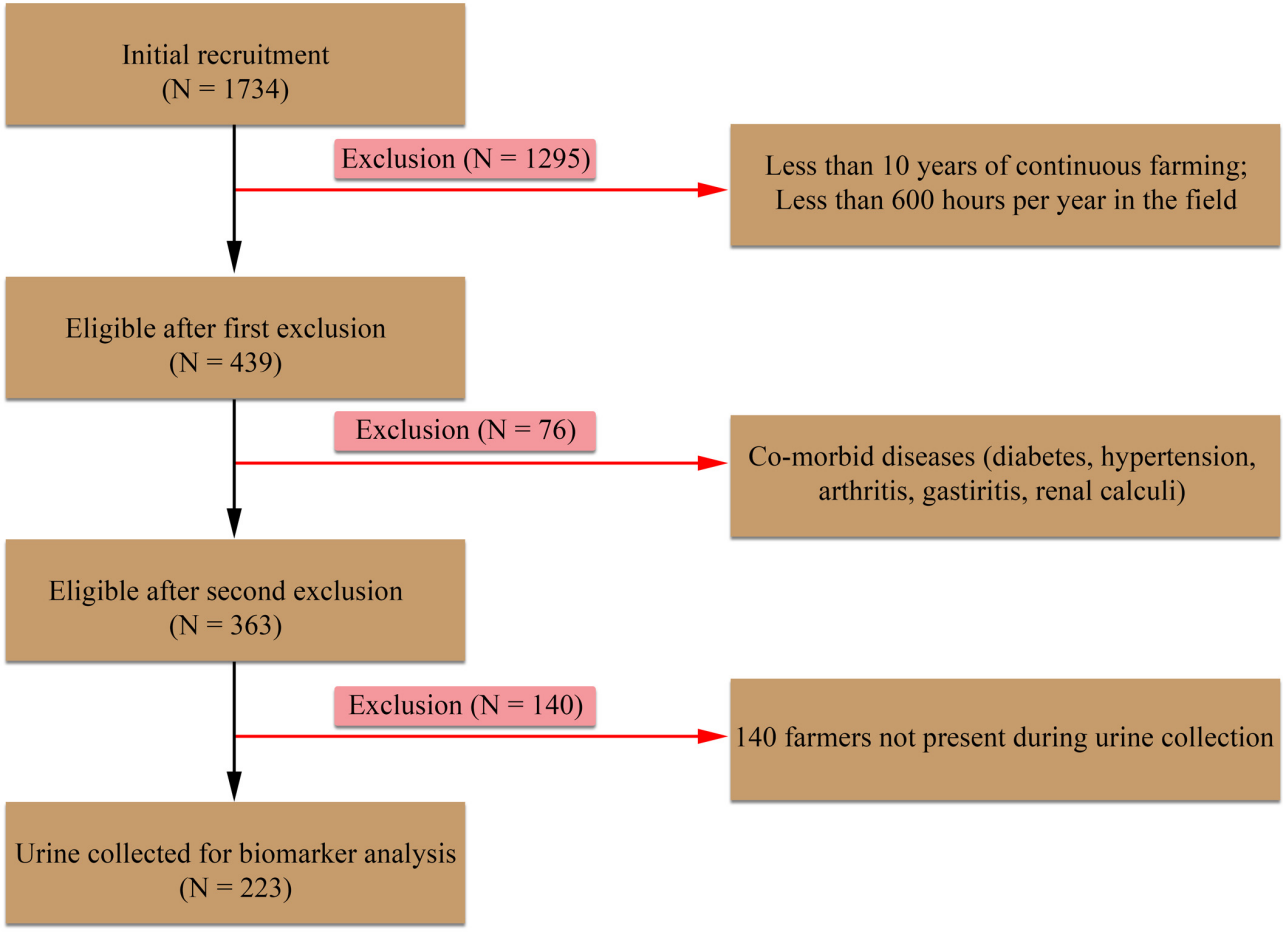
Flow chart representing study populations and study design in non-endemic control locations in Matara (CM), Nuwara Eliya (CN) and two farming locations (Agunukolapalassa—EL1 & Badagiriya—EL2).

Male farmers over 20 years (n = 1734) were recruited from all four selected farming locations. Participants were screened to exclude farmers with less than 10 years of farming and lower working hours (less than 600 hours per year). Therefore, based on above exclusion criteria 1295 participants were excluded and remaining 439 (EL1 = 106, EL2 = 127, CN = 104 and CM = 102) were screened for co-morbid diseases (Fig 2). An interviewer administered, pre-tested survey questionnaire was used to collect data from the farmers. During the interview, information about co-morbid diseases (i.e. diabetes, hypertension, arthritis, gastritis, renal calculi etc.) was obtained. 76 farmers had comorbidities and 363 farmers were selected for the study. However, 140 participants were absent during the urine collection and 223 urine samples EL1 (n = 56), EL 2 (n = 61), CN (n = 52) and CM (n = 54) were used for the biomedical analysis. Details of the study population were illustrated in (Fig 2).

### Ethics statement

Ethical approval was obtained from the ethics review committee of the Faculty of Medicine, Rajarata University, Sri Lanka. Farmers from all four locations were made aware about the study verbally and with information leaflets. A written consent (n = 1701) was obtained from each farmer. There were 33 farmers that were unable to write, in such cases thumb print consent was obtained following ethical guidelines. The study was conducted in accordance with Helsinki declaration.

### Measurement of creatinine in urine and serum

Urine and blood samples were collected from individuals to measure creatinine, eGFR, urine ACR, KIM-1, NGAL and HbA1c. Fresh morning first void urine samples were collected in sterile containers from each farmer and stored temporarily at 4°C. In the lab, urine samples were centrifuged for 15 minutes at 4000 rpm, 4°C and samples were stored in aliquots at -80°C until analysis. Blood samples were collected in labelled serum separator tubes and allowed to rest and clot at room temperature for 30 minutes. Blood sample tubes were then centrifuged at 2500 rpm for 10 minutes at 4°C. Later, the supernatant (serum) was labelled and transferred into cryogenic vials and stored at -80°C until analysis.

Creatinine was measured in urine and serum by modified kinetic Jaffe reaction to minimize interference of non-creatinine and jaffe-positive compounds [37, 38] in Dimension clinical chemistry system (Siemens, New York, U.S.A.). Picrate reacts with creatinine to produce a red chromophore in the presence of a strong base (NaOH). Absorbance was measured at 510 nm (assay range: 0 mg/dl– 20 mg/dl). Urinary or serum creatinine levels were expressed in mg/dl.

### Measurement of estimated Glomerular Filtration Rate (eGFR)

eGFR was calculated by using both CKD-EPI (CKD Epidemiology Collaboration) and modified MDRD equation [39]. GFR was expressed in mL/min/1.73 m^2^ of body surface area. A medical doctor in the study group measured the blood pressure after fifteen minutes’ rest, using a mercury sphygmo-manometer. The average of two readings taken five minutes apart was used.

### Measurement of microalbumin in urine

Microalbumin was measured in urine by particle-enhanced turbidimetric inhibition immunoassay (PETINIA) and Dimension clinical chemistry system (Siemens, Newark, U.S.A.). In the presence of human albumin bound particle reagent (PR), albumin present in the sample competes for monoclonal antibody (mAb) and reduces the rate of PR–mAb aggregation. Therefore, rate of aggregation was inversely proportional to albumin concentration in urine samples. Rate of aggregation was measured using bichromatic turbidimetric reading at 340 nm (assay range: 1.3 mg/L– 100 mg/L).

### Measurement of HbA1c

Ion-exchange high-performance liquid chromatography (HPLC) principle based separation of HbA1c on a cation exchange cartridge method was used for the measurement of HbA1c in human anti-coagulated whole blood samples. The absorbance of separated HbA1c was then measured at 415 nm using Bio-Rad D-10 as per manufacturer’s instructions.

### Measurement of human KIM-1 in urine samples

Human KIM-1 was measured in early morning urine samples using ELISA (CUSABIO, P.R. China; Cat#: CSB-E08807h) according to the manufacturer’s instructions. KIM-1 ELISA kit employs quantitative sandwich enzyme immunoassay technique for high sensitivity and specificity for human KIM-1 detection. Minimum detectable dose of human KIM-1 was typically less than 0.043 ng/ml. Intra-assay precision was (CV%: <8%) while inter-assay precision was (CV%: <10%). Detection range of the kit was (0.312 ng/ml—20 ng/ml). Absorbance was measured at 450 nm using micro plate reader (Utrao microplate reader–SM600, Shanghai Yong Chuang, P.R. China).

### Measurement of human NGAL/Lipocalin-2 in urine samples

Human NGAL/Lipocalin-2 was measured in early morning urine samples using ELISA (Ray Biotech, Inc. Norcross, GA; Cat: ELH-Lipocalin2-001) according to the manufacturer’s instructions. NGAL ELISA kit employs quantitative sandwich enzyme immunoassay technique for specific detection of human Lipocalin-2 or NGAL. Minimum detectable dose of human Lipocalin-2 was 4 pg/ml. NGAL ELISA kit intra-assay precision was (CV%: <10%) and inter-assay precision was (CV%: <12%). Detection range of the kit was (4 pg/ml—1000 pg/ml). Absorbance was measured at 450 nm using microplate reader (Utrao microplate reader—SM600, Shanghai Yong Chuang, P.R. China).

### Statistical analysis

Data were analysed using IBM statistics (version 22.0). Continuous variables were reported as means (SEM) whereas categorical variables were reported as proportions. Renal biomarkers (KIM-1 & NGAL) were adjusted for urine creatinine concentrations prior to analysis. All comparisons between groups were performed by one-way ANOVA test with normally distributed parameters or transformed to natural log parameters. Kruskall–Wallis test and the Mann–Whitney U-test were performed to compare the significance between groups when deviated from the normality. Association between renal biomarkers with Albumin- creatinine ratio (ACR) and eGFR were performed using linear regression models. In all analysis, P < 0.05 was considered as significant.

## Results

### Baseline characteristics of study sample

Baseline characteristics and information of the study sample were given in Table 1. Overall, 439 male farmers (age ≥ 20 years) participated in the cross-sectional study representing four different farming locations of Sri Lanka. Age of individuals ranged between 26–49 years in CM, 20–83 years in CN, 27–70 years in EL1 and 36–79 years in EL2. Most participants were paddy Farmers. Farmers from CN and CM were involved in vegetable farming in addition to paddy. Lower education level hence low socio—economic status has been reported in emerging locations EL1 and EL2 than CM and CN. The most significant factor was the previous source of drinking water where EL1 and EL2 farmers mainly consumed well water that has been categorized as very hard (≥ 181 ppm). However, most of the farming locations now have access to tap water. Co-morbid diseases (i.e. diabetes, hypertension, arthritis, gastritis, renal calculi etc.) were reported in certain farmers from all four farming locations with CM (4%), CN (11%), EL1 (27%) and EL2 (25%). Diabetes and hypertension were not reported within the study sample from CM. However, cases of diabetes and hypertension were reported within certain farmers from CN (1% and 6%), EL1 (6% and 12%) and EL2 (10% and 8%) respectively. Other co-morbid diseases such as arthritis, gastritis, renal calculi etc., were also reported in CM (4%), CN (4%), EL1 (9%) and EL2 (8%) respectively. Healthy subjects (i.e. no diabetes, hypertension, other kidney disease etc.) from CM, CN, EL1 and EL2 were 96% (n = 102), 89% (n = 104), 81% (n = 63) and 90% (n = 86) respectively.

**Table 1:** Baseline characteristics of the study populations representing non-endemic control groups (CM & CN), CKDu emerging locations Angunakolapelessa (EL1) and Bandagiriya (EL2). All variables are presented as % and numbers indicates medical conditions.

	Non-endemic locations		CKDu emerging locations	
	Matara (CM)	Nuwara Eliya (CN)	Angunakolapelessa (EL1)	Bandagiriya (EL2)
	(N = 102)	(N = 104)	(N = 106)	(N = 127)
**Age** (years ± S.E.M)	44.7 ± 1.8	48.9 ± 1.6	47.7 ± 0.9	56.8 ± 0.9
**Level of education (%)**				
Primary	15	13	34	44
Secondary/Higher	85	87	53	39
Uneducated	-	-	13	17
**Smoking (%)**	12	48	47	44
**Alcohol consumption (%)**	12	58	55	49
**Chewing betel (%)**	6	23	61	65
**Mosquito repellents (%)**	15	0	77	75
**Current source of drinking water (%)**				
Tap	91	82.6	62.2	99.2
Well	9	17.4	37.8	0.8
**Previous source of drinking water (%)**				
Tap	91	82.6	4.7	3.2
Well	9	17.4	95.3	96.8
**Pesticide usage (%)**	-	78	100	97
**Protection from pesticides (%)**	-	54	33	51
**Co-morbid diseases**				
Diabetes	-	1	6	12
Hypertension	-	6	13	10
Arthritis, Gastritis, renal calculi etc.	4	4	10	10

### Determination of CKDu cases based on WHO case definition

Albuminuria (ACR ≥ 30 mg/g Cr) in repeated occasions was not reported in both non-endemic locations (CM and CN) and therefore no CKDu cases were identified under WHO case definition. However, fourteen (18%) and nine (9%) individuals from emerging locations EL1 (Angunakolapelessa) and EL2 (Bandagiriya) were reported with repeated ACR ≥ 30 mg/g Cr. These cases were defined as CKDu patients and grouped into EL1-CKDu and EL2-CKDu in further biomarker analysis. The range reported was 31–124.4 mg/g Cr in EL1-CKDu and 35.2–82.2 mg/g Cr in EL2-CKDu. Healthy subjects (ACR ≤ 30 mg/g Cr) in EL 1 and EL 2 were considered as controls from the emerging locations and grouped into C-EL1 and C-EL2. ACR of farmers from CM, CN, C-EL1 and C-EL2 was not significant with each other (p>0.05) except isolated two CKDu groups. Mean eGFR in both CM and CN was 108 ml/mi/1.73m^2^ and slightly lower eGFR was recorded at emerging locations EL1 and EL2. The lowest eGFR was recorded in EL2-CKDu. No cases of eGFR ≤ 60 ml/min/1.73m^2^, were reported in non-endemic locations (CM & CN) but three individuals (4%) from EL1 and two individuals (2%) from EL2 had eGFR ≤ 60 ml/min/1.73m^2^ Table 2.

**Table 2:** Clinical characteristics of the study populations representing non-endemic locations in Matara (CM), Nuwara Eliya (CN) and CKDu emerging locations Angunakolapelessa (EL1) and Bandagiriya (EL2).

	Non-endemic locations		CKDu emerging locations	
	Matara (CM)	Nuwara Eliya (CN)	Angunakolapelessa (EL1)	Bandagiriya (EL2)
	(N = 98)	(N = 93)	(N = 77)	(N = 95)
**ACR (mg/g)**				
Mean ±S.E.M	3.2±0.4	8.6±0.8	17.4±2.4	13±1.5
**ACR ≥ 30 mg/g**	00 (0%)	00 (0%)	14 (18.18%)	09 (9.47%)
**Serum creatinine (mg/dL)**				
Mean ±S.E.M	0.89±0.03	0.79±0.001	0.93±0.02	0.93±0.01
**eGFR (ml/min/1.73 m**^**2**^**)**				
CKD-EPI; Mean ±S.E.M	110±2.6	107±1.3	98±1.7	92±1.5
MDRD; Mean ±S.E.M	103±3.2	107±1.5	91±1.8	90±1.9
**eGFR ≥ 60 ml/min/1.73m**^**2**^	98/98 (100%)	93/93 (100%)	74/77 (96%)	93/95 (98%)
**eGFR ≤ 60 ml/min/1.73m**^**2**^	00 (0%)	00 (0%)	03 (4%)	02 (2%)

### Urinary biomarker analysis for kidney injury

**Fig 3:**
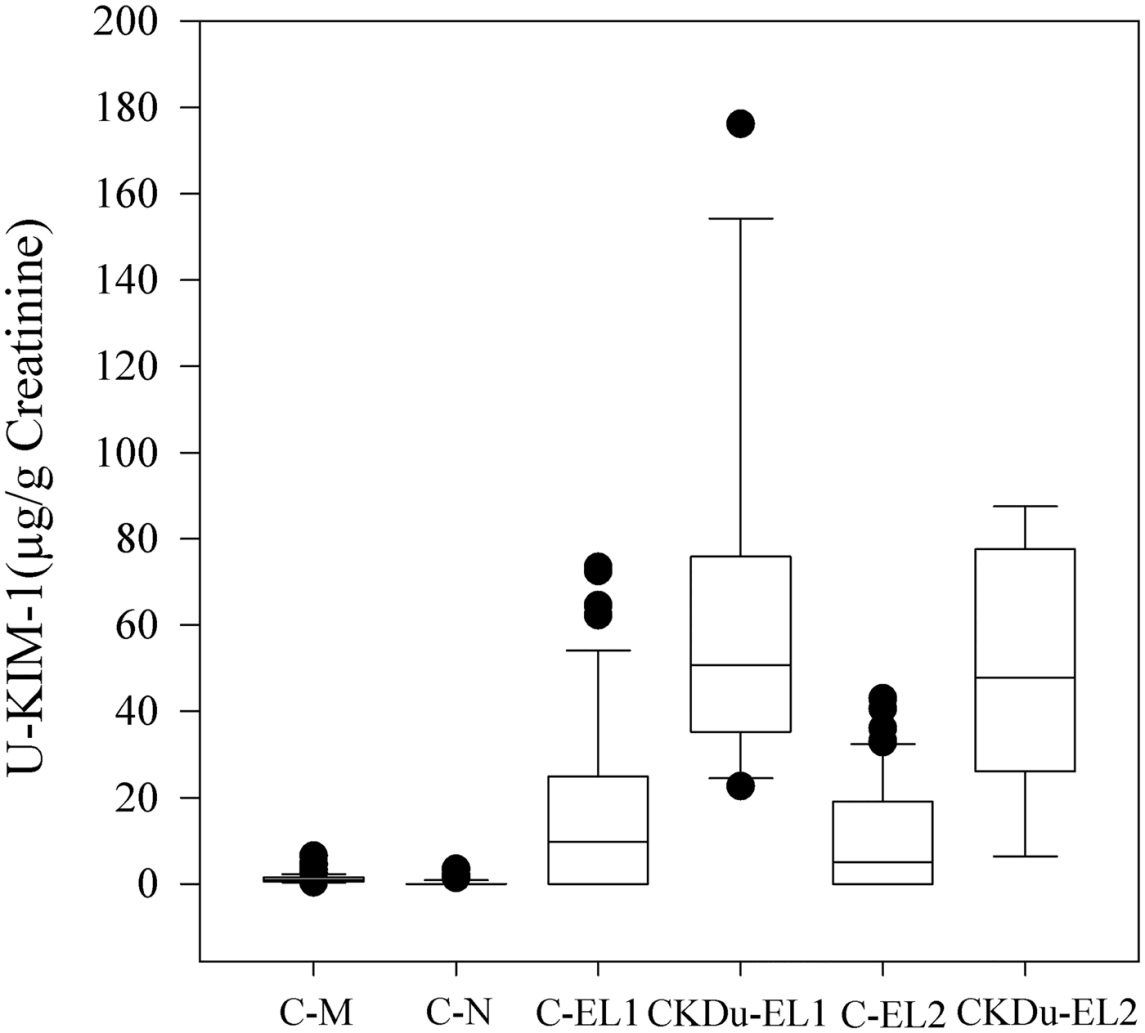
Mean urinary KIM-1 (μg/g Cr) in the control groups (CM & CN), CKDu groups (EL1-CKDu & EL2-CKDu) with control groups from CKDu emerging locations (C-EL1 & C-El2).

Summarised statistics of KIM-1 normalized to urinary creatinine along with respective ACR, eGFR and HbA_1C_ are presented in Table 3. The lowest mean values of urinary KIM-1 were reported in CN (0.17 μg/g Cr) followed by non-endemic controls in CM (1.26 μg/g Cr). Higher urinary KIM-1 was reported in CKDu emerging locations EL-1 and EL–2 with compared to both CM and CN. The highest KIM-1 levels (64.27 μg/g Cr and 48.53 μg/g Cr) were reported by CKDu subjects in both EL-1 and EL-2. As seen in Fig 3, urine-KIM-1 concomitantly increased in CKDu cases identified in farming locations (EL1- CKDu and EL2-CKDu). KIM-1 values reported at EL1-CKDu and EL2-CKDu were 50 fold and 38 fold higher than the control farming location (CM). They also reported significantly higher concentrations in comparison with CN (P < 0.001). Higher KIM-1 was also reported in healthy farmers (ACR ≤ 30 mg/g Cr) at EL1 and EL2. The highest urinary KIM-1 (16.07 μg/g Cr) recorded in C-EL1 was twelve times higher than the control farming location (CM). However, a lower KIM-1 concentration (10.52 μg/g Cr) was recorded at farming location C-EL2. KIM-1 levels in C-EL1 and C-EL2 were also significantly higher than the KIM-1 levels in non-endemic farming controls in CN (P < 0.001).

**Table 3:** Creatinine adjusted tubular markers (KIM-1 & NGAL) and clinical data in non-endemic controls (CM & CN) and two CKDu emerging locations (EL1 and EL2). EL1 and EL2 further divided as control subjects from the same locations (C-EL1 & C-EL2) and CKDu subjects (EL1-CKDu & EL2- CKDu). All variables are present as mean (SEM) and range.

	Non-endemic locations		Emerging location 1 (EL1)		Emerging location 2 (EL2)	
	CM (N = 54)	CN (N = 52)	C-EL1 (N = 42)	EL1 –CKDu (N = 14)	C-EL2 (N = 52)	EL2 –CKDu (N = 09)
**KIM-1 (μg/g Cr)**	1.26 (0.15)	0.17 (0.08)*	16.07 (3.19)* ^†^	64.27 (11.43)* ^†^	10.52 (1.72)* ^†^	48.53 (9.37)* ^†^
	0.3–6.46	0–3.4	0–73.39	22.55–176.11	0–42.90	6.41–87.48
**NGAL (μg/g Cr)**	0.30 (0.03)	0.47(0.05)	0.76 (0.11)	2.46 (0.60)* ^†^	1.16 (0.09)	6.95 (2.44)* ^†^
	0.02–0.97	0–1.63	0–2.69	0.16–8.78	0.18–2.84	0.45–24.12
**ACR (mg/g Cr)**	3.21 (0.48)	8.85(1.1)	16.14 (1.7)	52.89 (8.77)	9.82 (1.57)	54.26 (6.09)
	1.16–13.00	2.95–27.3	2.6–29.4	31–124.4	0.6–28.2	35.2–82.2
**Serum creatinine (mg/dL)**	0.9(0.03)	0.8(0.01)	0.9(0.04)	0.9(0.03)	0.9(0.04)	1.1(0.06)
**eGFR (ml/min/1.73m**^**2**^**)**	108 (2.5)	108 (1.88)	95.81 (4.51)	96.5 (3.76)	94.93 (4.93)	78.78 (5.85)
	92–134	94–134	58–128	72–121	47–134	57–111
**HbA1c (%)**	–	–	–	5.51 (0.10)	–	5.72 (0.15)
				5.1–5.8		5.0–6.5

* Indicates P < 0.05 vs. non endemic control (CM)

^**†**^ indicates P < 0.05 vs. non endemic control (CN).

**Fig 4:**
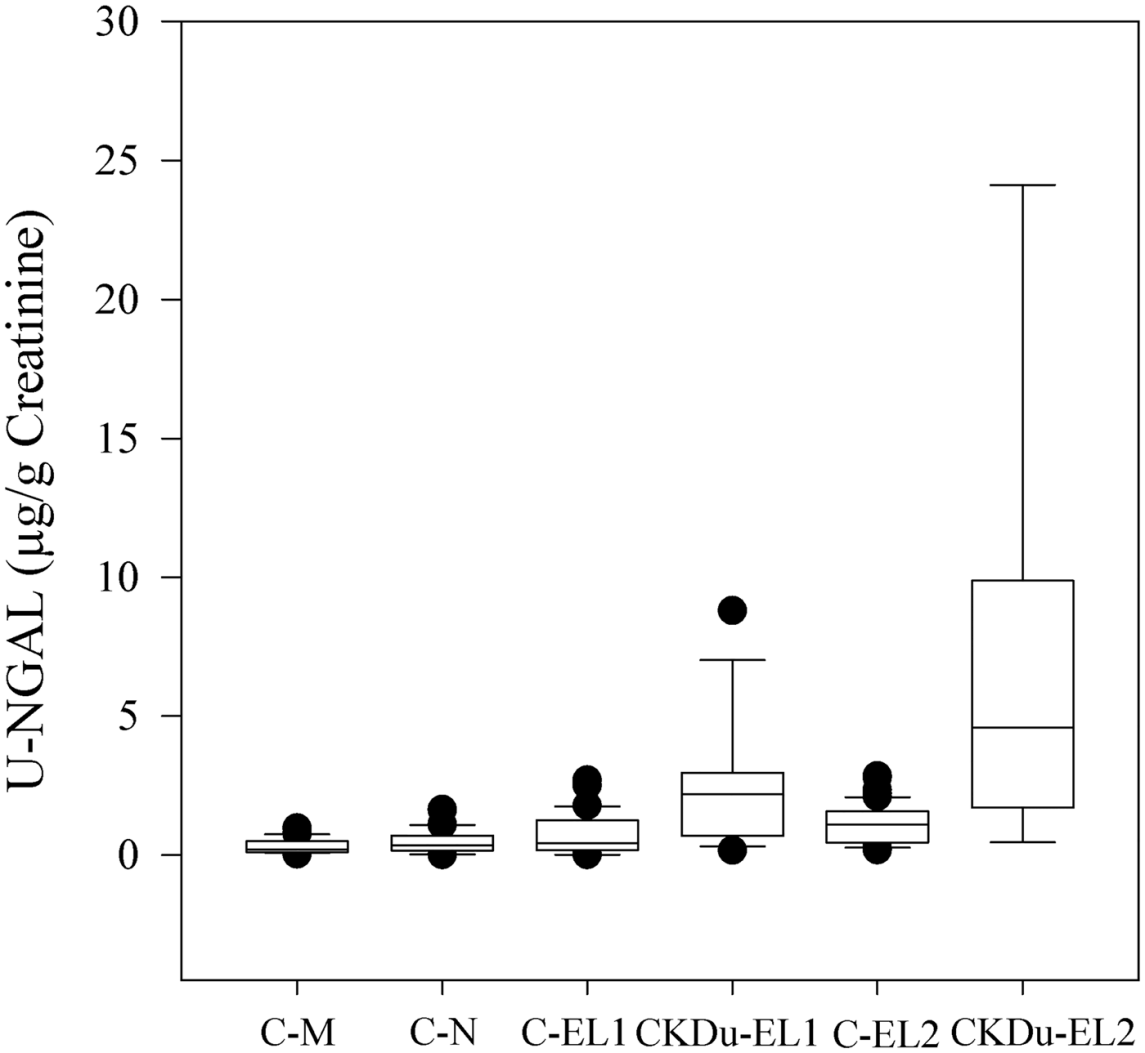
Mean urinary NGAL (μg/g Cr) in the control groups (CM & CN), CKDu groups (EL1-CKDu & EL2-CKDu) with control groups from CKDu emerging locations (C-EL1 & C-El2).

Creatinine adjusted NGAL levels in non-endemic farming locations (CM, CN) and emerging farming locations (EL1 and EL2) were given in Table 3. The lowest mean NGAL (0.30 μg/g Cr) was reported in non- endemic farmers in Matara (CM) followed by non-endemic farmers in Nuwara Eliya (CN; 0.47 μg/g Cr). Both CKDu groups in emerging locations, EL1-CKDu (2.46 μg/g Cr) and EL2-CKDu (6.95 μg/g Cr) reported the highest NGAL. Pairwise comparisons revealed that NGAL levels at both CKDu groups were significantly higher than the control farming locations (CM & CN, P < 0.001; Fig 4). NGAL levels of C -EL1 and C- EL2 were approximately 2.5 fold and 4 fold higher than the control group CM and 1.6 fold and 2.4 fold higher than the CN. However, NGAL levels in C -EL1 and C- EL2 were not significant (P > 0.05) with comparison to both control groups CM and CN. CKDu cases in both emerging locations (EL1-CKDu and EL2-CKDu) were also reported significantly higher NGAL when compare to healthy subjects (C -EL1 & C- EL2) in the same location (P < 0.001).

**Fig 5:**
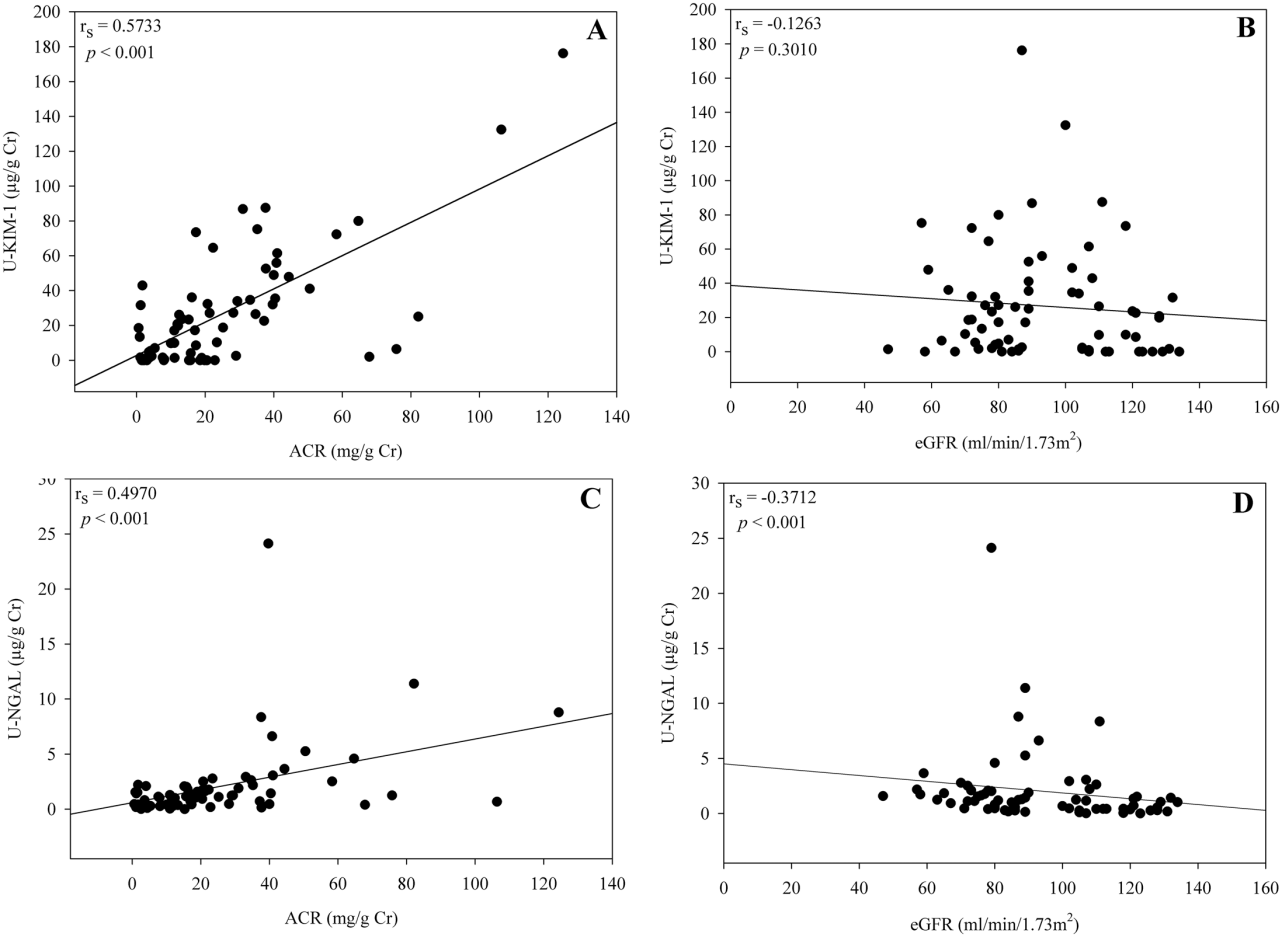
Association of albumin to creatinine ratio (ACR, mg/g Cr) with urinary KIM-1 (5A), estimated glomerular filtration rate (eGFR) (ml/min/1.73 m^2^) with urinary KIM-1 (5B), albumin to creatinine ratio (ACR, mg/g Cr) with urinary neutrophil gelatinase-associated lipocalin (5C) and estimated glomerular filtration rate (eGFR) (ml/min/1.73 m^2^) with urinary NGAL (5D) in the CKDu emerging locations EL1 and EL2.

Analysis of urinary biomarkers (KIM-1 and NGAL) following the isolation of CKDu cases (based on urinary albumin-to-creatinine ratio (ACR) ≥ 30 mg/g Cr) revealed a significant correlation between increased urinary renal biomarker levels and albuminuria. Elevated levels of urinary KIM-1 were positively correlated with increased ACR in EL1 and EL2 (r_s_ = 0.57, P < 0.001; Fig 5A) however, no significant correlation was found between Urinary KIM-1 and eGFR (r_s_ = -0.12, P = 0.30; Fig 5B). Similarly, elevated levels of urinary NGAL was positively correlated with increased ACR in CKDu emerging locations (EL1 and EL2) (r_s_ = 0.49, P < 0.001; Fig 5C) and significant negative correlation was observed between urinary NGAL and eGFR in EL1 and EL2 (r_s_ = -0.37, P < 0.001; Fig 5D).

## Discussion

The current study contributes to reveal the role of novel urinary biomarkers (KIM-1 and NGAL) in CKDu detection for the first time in Sri Lanka. This is also the first cross-sectional study exploring chronic kidney disease of uncertain etiology (CKDu) adopting WHO guidelines based CKDu case definition in Hambantota district, Southern Province, Sri Lanka. No studies have been previously reported using combination of urinary ACR, KIM-1 and NGAL together in determining early CKDu cases among Sri Lankan farming community. New CKDu cases (6%, 23/363) were identified during the study based on WHO study group definition. Albuminuric groups (EL 1-CKDu & EL 2 –CKDu) reporting the highest, non-endemic control subjects (CM & CN) showing the lowest and healthy subjects in emerging locations (C-EL1 &C-EL 2) showing intermediate values of KIM -1 and NGAL indicate occurrence of sub-clinical renal injury. A gradient with clear separation of KIM -1 and NGAL values in albuminuric, non-endemic controls and endemic controls was evident. Overall, higher levels of urinary KIM-1 may indicate the proximal tubular damage whereas higher levels of urinary NGAL might be likely due to detectable damage occurring in loop of Henle and distal convoluted tubule.

In Sri Lanka, men are more vulnerable to CKDu than women. A study conducted in NCP showed CKDu prevalence is higher in males (6%) than in females (2.9%) [1]. Similarly, in El Salvador, the prevalence of CKDu was reported higher in men (25.7%) than in women (11.8%) [4]. A meta-analysis based investigation using 68 studies concluded that males with non-diabetic renal disease showed significantly rapid kidney function deterioration over time than females [40]. Rapid progression of males from early stages of renal damage to chronic stages of kidney injury was most probably due constant exposure towards occupational or environmental factors [3–5, 26]. As a consequence, the current study was precisely focused on male farmers excluding females and children. In 2011, a single suspected case of CKDu (0.025%) was reported in Hambantota district despite it was previously considered as a non-endemic region [27]. In the same year, six (0.43%) CKDu cases were identified in Hambantota district using non-specific qualitative dipstick test followed by sulfosalicylic acid test [1]. Hambantota, located in the dry zone, shares similar socio-economic background and identical farming practices to the CKDu endemic NCP in Sri Lanka. Therefore, there is a looming possibility of emergence of CKDu in Hambantota district, Southern Province, Sri Lanka.

Here, we report distinct 23 CKDu cases from EL1 and EL2 in Hambantota district, Southern Province, Sri Lanka, based on both CKDu definition by WHO [26] and increased levels of KIM-1and NGAL. Co-morbid diseases (i.e. diabetes, hypertension, pyelonephritis, renal calculi etc.) may influence levels of urinary ACR, KIM-1 and NGAL [41–45]. Consequently, we utilized questionnaire and medical history of individuals based assessment to identify and eliminate cases with co-morbid diseases. HbA1c was also measured in individuals with ACR ≥ 30 mg/g in EL1 and EL2 to exclude diabetes cases. Therefore, reported 23 new cases can be confirmed as CKDu. All CKDu cases (EL1-CKDu and EL2-CKDu) had ACR ≥ 30 mg/g on repeated testing. Measurement of albumin levels is a well-known early non-invasive biomarker to detect CKD [46, 47]. An epidemiological study also supports the notion of testing albuminuria as an indicator of renal disease in general population [48]. Urinary albumin levels define the glomerular integrity and proximal tubule function in kidneys [49].

CKDu cases were also confirmed by significantly higher levels of urinary KIM-1 and NGAL. Both markers could easily isolate the suspected cases. Apparently healthy farmers at emerging locations (C -EL1 & C- EL2) who had ACR < 30 mg/g, healthy eGFR and normal serum creatinine also showed elevated levels of KIM-1 and NGAL compared to both non-endemic control groups (CM & CN) indicating possible early renal damage. It may suggest that tubular damage expressed by KIM-1 is present before albuminuria appeared in farmers from C -EL1 & C- EL2 Similar cases have been reported among sugarcane cutters in Nicaragua with elevated urinary NGAL, IL-18 and NAG [30]. Urinary KIM-1 detection using micro-urine nanoparticle detection technique has been recently reported in Sri Lanka [50] but comparison is not possible due to smaller sample size of the study. KIM-1 is markedly up regulated in kidneys due to ischemic insult [35, 51]. Up regulation of KIM-1 is a well-known consequence of proximal tubular damage in the nephron. Until more recently, detecting glomerular KIM-1 expression could also be a useful tool in identifying glomerular injury [52]. Increased levels of KIM-1 may also represent its involvement in phagocytosis of damaged proximal tubule epithelial cells by converting epithelial cells into semi-professional phagocytes [53, 54]. KIM-1 up regulation may also be responsible in restoring functional and morphological integrity of kidneys following ischemic insult [35]. Our study shows that KIM-1 may be used to detect early CKDu cases in susceptible farming communities in Sri Lanka other than the conventional markers.

However, NGAL elevation was only notable in EL1-CKDu and EL2-CKDu with 8 fold and 23-fold increase with compared to CM and 5 fold and 14-fold increase with CN. Similar results have been reported in El-Salvador where 26% higher NGAL was reported in CKD cases [55]. Laws et al., reported 1.49 times higher NGAL among sugarcane farmers in Nicaragua [30]. However, no studies have been reported using NGAL in Sri Lankan population. NGAL elevation suggest, re-epithelialisation of damaged tubules and reabsorption of iron that was leaked due to damage of proximal epithelial tubule cells and also to induce iron-dependent nephrogenesis [51, 56]. NGAL was not significantly increased in C—EL1 & C—EL2 when compared to CM and CN. This suggests elevation of KIM-1 may be more sensitive in detecting early tubular damage when compared with NGAL. Therefore, this study does not support the use of NGAL to detect early cases of CKDu in susceptible populations however, further studies are necessary.

There are some limitations in our study. We initially recruited 1734 farmers. We used precise inclusion criteria to limit the study population i.e., continuous farming (> 10 years) with long working hours (> 600 hours per year). These inclusion criteria at the beginning of the study lead to a smaller sample size (n = 439). Some farmers (n = 140, 38.6%) were not present at the time of urine collection therefore the study left with modest sample size for the biomarker analysis (n = 223). Other main limitation of the study was lack of established urinary biomarker levels that reflect sub clinical damage in Sri Lankan nephropathy. No previous studies have been done under local conditions and comparable occupational cohorts are even difficult to find in Mesoamerican nephropathy except a few recent studies [30, 31]. Short term individual variation within the subjects was also unknown and a follow up study is required. The current study was conducted only on native male farmers in selected farming locations in Sri Lanka ignoring children and females therefore might hinder generalized applicability of the findings in other geographical locations and general population.

In conclusion, this study reports 23 new CKDu cases for the first time in Hambantota district, Sri Lanka in spite of previously being considered as a non-endemic location. This is the first study to identify CKDu suspected cases and detection of early kidney damage in Sri Lankan farming communities using urinary biomarkers KIM-1 and NGAL. New cases were defined by WHO study group criteria for CKDu diagnosis in Sri Lanka along with urinary markers KIM-1 and NGAL. The results of our cross-sectional study shows that the tubular damage predicted by urinary KIM-1 and NGAL were significantly correlated with high urinary ACR levels. Strikingly, early tubular damage as seen by higher urinary KIM-1 and NGAL was also observed in healthy farmers despite normal ACR levels (< 30 mg/g). Urinary tubular markers reconfirm tubulointerstitial disease with repeated tubular injury in CKDu among farming communities in Sri Lanka. However, longitudinal cohort studies are needed to predict use of tubular markers for precise prognosis, optimized treatment and patient management.

## Supporting Information

S1 File STROBE statement. (DOC)
